# Using network analysis to study behavioural phenotypes: an example using domestic dogs

**DOI:** 10.1098/rsos.160268

**Published:** 2016-10-19

**Authors:** Conor Goold, Judit Vas, Christine Olsen, Ruth C. Newberry

**Affiliations:** 1 Department of Animal and Aquacultural Sciences, Norwegian University of Life Sciences, Ås, Norway; 2 Section of Public Health, Department of Landscape, Architecture and Spatial Planning, Norwegian University of Life Sciences, Ås, Norway

**Keywords:** phenotypic integration, network analysis, dog behaviour, personality, self-organization, play behaviour

## Abstract

Phenotypic integration describes the complex interrelationships between organismal traits, traditionally focusing on morphology. Recently, research has sought to represent behavioural phenotypes as composed of quasi-independent latent traits. Concurrently, psychologists have opposed latent variable interpretations of human behaviour, proposing instead a network perspective envisaging interrelationships between behaviours as emerging from causal dependencies. Network analysis could also be applied to understand integrated behavioural phenotypes in animals. Here, we assimilate this cross-disciplinary progression of ideas by demonstrating the use of network analysis on survey data collected on behavioural and motivational characteristics of police patrol and detection dogs (*Canis lupus familiaris*). Networks of conditional independence relationships illustrated a number of functional connections between descriptors, which varied between dog types. The most central descriptors denoted desirable characteristics in both patrol and detection dog networks, with ‘Playful’ being widely correlated and possessing mediating relationships between descriptors. Bootstrap analyses revealed the stability of network results. We discuss the results in relation to previous research on dog personality, and benefits of using network analysis to study behavioural phenotypes. We conclude that a network perspective offers widespread opportunities for advancing the understanding of phenotypic integration in animal behaviour.

## 1. Introduction

Understanding the biological organization of complex phenotypes is a mainstay of evolutionary biology [1–3]. Phenotypic integration describes the ‘pattern of functional, developmental and/or genetic correlation…among different traits in a given organism’ [4, p. 266]. Most commonly, phenotypic integration has been concerned with morphological traits (e.g. beak length and size in Darwin's finches [5]; sexual traits [6]). Recently, organization of the behavioural phenotype has also been cast in terms of phenotypic integration. Araya-Ajoy & Dingemanse [7], inspired by research in human psychology, propose that behavioural phenotypes consist of a collection of latent variables (behavioural *characters*) that play a causal role in producing correlated responses in patterns of behaviour, both within and between individuals. They discuss how this conceptualization could be applied to a number of topical themes in the study of animal behaviour including personality (consistent between-individual differences in behaviour) [8] and behavioural plasticity (between-individual differences in behavioural change) [9].

Phenotypic integration of biological traits is increasingly envisaged as interactions (whether physical or correlational) between modules played out on complex networks [10]. For example, Perez *et al.* [11] demonstrate how landmarks of the mammalian mandible can be represented as a network of nodes and correlational edges. Moreover, Wilkins *et al.* [6] advocate a ‘phenotype network’ approach for understanding correlations between sexual traits in the North American barn swallow (*Hirundo rustica erythrogaster*). The merit of a network perspective is that it naturally incorporates interactions within the components of, and between different, functional traits (‘trait complexes’) [12] and provides novel analytical insights (e.g. global and local network metrics). This is commensurate with studying organisms as ‘developmentally, functionally and phenotypically integrated complex units’ [13, p. 279], which numerous researchers argue is integral to improving our knowledge of the phenotype [3,14–16]. It follows that the organization of the behavioural phenotype can also benefit from being represented as an integrated network.

A network perspective has recently emerged in human psychology [17]. Psychological phenomena, such as personality dimensions (e.g. the Five Factor Model) [18], have traditionally been represented as latent variables and analysed with principal components analysis or varieties of factor analysis, respectively. However, this latent variable formulation has been contested (e.g. see commentaries in [19]), based on long-standing concerns that latent variable approaches can be conceptually, statistically and empirically ambiguous [20–24]. The central criticisms are that: (i) latent variables are often represented as fixed entities, failing to portray the dynamics of individual patterns of behaviours and the variability or lack of unidimensionality in psychological variables [23,25,26], (ii) observed behaviours are treated as passive and exchangeable indicators of the particular latent state [27,28], (iii) finding realizations of latent variables in biological organization (e.g. intelligence) [22] is challenging and, more conceptually, (iv) latent variables are unobservable by definition [28,29], promoting circularity in definitions of psychological phenomena (‘verbal magic’) [21] and leading to the fallacy of misplaced concreteness [30].

The network approach expounded by Cramer *et al.* [17,19] (see also [31–33]) presents personality and psychopathological phenomena as networks of autonomous and causally related cognitive, affective and behavioural components. These components possess conditional independence relationships, such that variation in one component can result in variation in another component conditional on all other measured components [34,35]. Given this assumption, components are more likely to have causal relationships when they possess a functional relationship, and when multiple components form close connections, functional clusters may emerge. For instance, networks of symptoms (e.g. ‘loss of energy’ and ‘weight/appetite change’) in long-term patients with major depression disorder were more densely connected (i.e. had greater network connectivity) than those of remitted patients [27]. Van der Mass *et al.* [22] further show how the positive manifold of general intelligence, defined as the observed correlations between cognitive skills related to intelligence, can be explained (and predicted) by direct mutualistic feedback relationships between those cognitive skills. While relationships between network components are influenced by underlying biological mechanisms (e.g. developmental pathways or genetic covariance [36,37]), the network approach aims to understand the behavioural phenotype as its own causal network of self-organizing components, rather than being comprised of passive indicators of ‘common cause’ latent variables [17].

In this paper, we synthesize the themes introduced above by exploring direct relationships among different behavioural and motivational characteristics in domestic dogs (*Canis lupus familiaris*). Dogs are useful in this respect because it is possible to gather information efficiently about multiple variables in a range of contexts using surveys directed at dog owners, who interact with their dog on a regular basis and are thus qualified to answer questions about their dog's typical behaviour. Such surveys have been shown to be reproducible and corroborate behavioural observations (e.g. clinical reports) [38]. Until now, multivariate data on dog behaviour (e.g. from surveys or direct behavioural assessments) have usually been analysed using latent variable methods to reduce dimensionality and extract latent behavioural traits, or dimensions of dog personality, that explain the correlations between measured variables. This approach has resulted in the identification of a wide number of possible traits [38–40]. Alas, these putative traits often lack strong predictive validity [41–43], a practical concern when recruitment of suitable dogs for specific human uses depends upon reliable predictions. One possible reason is that predictive power is diminished when traits are overestimated as stable, dissociated constructs rather than components of dynamic integrated phenotypes. Further, after conducting a meta-analysis on behavioural consistency across numerous traits, Fratkin *et al.* [40] emphasized that personality dimensions in dogs may still be changeable in adults and sensitive to environmental and social perturbations. Thus, network analysis may be particularly beneficial when applied to the study of dog behaviour because it takes a bottom-up perspective to analysing direct functional relationships between behavioural components rather than decomposing the phenotype into latent variables.

Below, we apply network analysis to survey data collected from police dog handlers on desirable and undesirable behavioural and motivational descriptors of police patrol and detection dogs. Patrol dogs are selected and trained for diverse tasks, such as patrolling areas, controlling crowds, and tracking and detaining suspects, whereas detection dogs search for contraband, commonly drugs and money. Although studies have explored differences between working and non-working dogs on broad behavioural dimensions [44,45], few have compared different types of working dogs. A better understanding of how police dog behaviour is organized is of practical relevance to dog recruitment and training for specialized duties. Rather than focusing on deriving assumed latent traits as a basis for predicting future performance, we elucidate network structures that represent the behavioural phenotypes of patrol and detection dogs. Although our analyses are primarily exploratory, we expected to find some differences between patrol and detection dog networks due to differences in working duties. To our knowledge, this is the first application of network analysis to understand the behavioural phenotypes of animals.

## 2. Material and methods

### 2.1. Subjects

This study was carried out in collaboration with members of the Norwegian Police University College in Kongsvinger, Norway who oversee dog selection and training for the Norwegian police force. Professional police dog handlers (*N* = 227) across Norway were invited to complete an online survey in Norwegian investigating the personality and performance of police dogs. Handlers were requested to fill out one survey for each adult dog they had worked with as a handler. A total of 174 surveys were submitted. Three were removed for pertaining to more than one dog. The remaining responses concerned 171 dogs from 117 handlers (mean ± s.d. survey response per person: 1.46 ± 0.65), including 117 patrol dogs (91 German shepherd dogs; 22 Belgian malinois; 1 rottweiler; 1 giant schnauzer; 1 Belgian tervueren; 1 unrecorded breed) and 54 detection dogs (17 labradors; 12 flat coated retrievers; 8 German shepherd dogs; 8 springer spaniels; 2 Belgian malinois; 2 Welsh springer spaniels; 1 German shepherd dog × Belgian shepherd dog; 1 labrador × German pointer; 1 cocker spaniel; 1 Nova Scotia duck-tolling retriever; 1 unrecorded breed). Breed differences were not explored due to the limiting sample sizes. Dogs were mostly entire (*n* = 117) and male (*n* = 149). Responses were received from 79 male and 17 female handlers (21 did not disclose their sex), aged between 28 and 57 (28–37 years: *n* = 18; 38–47 years: *n* = 50; 48–57 years: *n* = 28; undisclosed: *n* = 21)*.* Handlers had between 1 and 30 years of experience as police dog handlers, and on average had 3.75 (s.d. = 4.64) previous dogs (including pet and working dogs).

### 2.2. Survey development

Survey questions and instructions were constructed in English, translated to Norwegian and back-translated to English to confirm intended meanings. The ‘personality section’ of the survey included 43 situational and adjective-based descriptors of police dog behavioural characteristics (electronic supplementary material, table S1). The list of descriptors was developed through (i) discussion with members of the Norwegian Police University College to include desirable and undesirable behavioural characteristics of relevance to police dog handlers, (ii) incorporation of characteristics evaluated in standardized assessments of Norwegian police dog behaviour, and (iii) refinement following pilot tests for comprehensibility. Dog handlers rated how well they agreed with the descriptors as portrayals of their dog's typical behaviour, which ranged from 1 = ‘Strongly disagree’ to 5 = ‘Strongly agree’, where 3 = ‘Neutral’. Participants could also choose 0 = ‘Not relevant/I do not know’. All participants were familiar with the terminology used as descriptions of police dog behaviour.

### 2.3. Data preparation

All data handling and analysis was conducted using R v. 3.2.3 [46] (see the electronic supplementary files for the R script). The raw data for each descriptor contained a mean ± s.d. of 1.23 ± 1.38 (0.72 ± 0.81%) truly missing responses and 4.16 ± 7.64 (2.43 ± 4.47%) zero responses (‘Not relevant/I do not know’). Zero responses were particularly prevalent for certain descriptors and five descriptors with at least 10% of zero responses were removed (electronic supplementary material, table S1). The remaining 38 descriptors were all of relevance to both dog types. One handler's responses for a patrol dog were removed as 18.5% were coded as zero (after removal of the five descriptors above), whereas the mean ± s.d. of the percentage of zero responses per dog was 1 ± 2.6%. The remaining zero responses were converted to missing values (as these were not comparable to other responses on the 1–5 scale).

### 2.4. Multiple imputation

Subsequently, a multiple imputation procedure (using *Amelia*) [47] was used to impute missing scores, rather than applying listwise deletion or mean substitution [48–50]. To ensure its robustness, we investigated any further biases in the data. We first considered whether the pattern of missingness in the data was dependent on dog type (i.e. patrol dogs and detection dogs), or on handlers for those submitting multiple surveys on different dogs (see §1 of the electronic supplementary material for statistical details). There were fewer missing values in the patrol than detection dog responses, and differences in the number of missing values varied between handlers. Thus, we included dog type and numerical handler ID as relevant conditioning variables for the multiple imputation procedure. Secondly, we investigated whether any descriptors had too many missing values to impute. The proportion of missing responses advisable for multiple imputation procedures is variable [51], although 5% or less is commonly considered unproblematic whereas greater than 5% [52] or 10% [53] have been reported to bias results. We chose to remove four descriptors with greater than 5% of missing responses (electronic supplementary material, table S1). Finally, we identified five pairs of variables that were theoretically similar and had high correlations relative to the data as a whole (polychoric correlations > |0.8|; see §2 of the electronic supplementary material for details), indicating redundancy. Therefore, we removed one descriptor from each pair (retaining the more specific one where evident, on the presumption that it was answered more reliably; electronic supplementary material, table S1). The resulting 29 descriptors had a mean of 1.2 ± 1.03% missing responses.

Subsequently, 15 multiply imputed datasets were generated. We averaged the datasets and rounded any non-integers to integers to produce a single dataset of ordinal responses. We examined the independence of responses to each question by the 44 handlers who filled out surveys for more than one dog. For eight descriptors, a high ratio of between- to within-handler variation indicated that repeated responses by the same handler lacked independence (see §3 of the electronic supplementary material for methods). Therefore, these eight descriptors were removed (electronic supplementary material, tables S1 and S2). Because the descriptor ‘Good at catching a ball’ had a particularly low variation ratio (defined as the proportion of responses not the mode) relative to other descriptors (mode = 5; variation ratio = 0.124), it was also removed. The final 20 descriptors used for the network analyses are presented in table 1, along with their modes, variation ratios and abbreviations used in the figures below.

**Table 1.** Descriptors used in the network analysis, including their abbreviations, modes and variation ratios (whole sample statistics shown outside parentheses; patrol and detection dog statistics, respectively, shown within parentheses). Descriptors are placed in alphabetical order (see electronic supplementary material, table S1, for ordering used in the survey).

abbreviation	descriptor name	mode	variation ratio
ACT^a^	active and nimble	5 (5; 5)	0.247 (0.284; 0.167)
ADP^a^	adapts to new situations quickly	5 (5; 5)	0.406 (0.414; 0.389)
CUR^a^	curious	5 (5; 5)	0.229 (0.224; 0.241)
DA^b^	aggressive towards other dogs (‘Dog aggressive’)^c^	4 (4; 1)	0.735 (0.707; 0.685)
FDA^b^	guards food (‘Food aggressive’)	1 (1; 1)	0.418 (0.414; 0.426)
FIT^a^	physically fit	5 (5; 5)	0.247 (0.293; 0.148)
FL^a^	fearless	5 (5; 5)	0.482 (0.422; 0.611)
FoH^b^	fear of heights	1 (1; 1)	0.461 (0.457; 0.500)
FSH^a^	able to stay focused during searches	5 (5; 5)	0.324 (0.371; 0.222)
GUS^b^	gives up searches quickly	1 (1; 1)	0.553 (0.586; 0.481)
GWL^b^	strong tendency to growl at strangers	1 (1; 1)	0.476 (0.483; 0.463)
PLA^a^	playful	5 (5; 5)	0.200 (0.207; 0.185)
PS^a^	solves problems on own (‘Problem solving’)	5 (5; 5)	0.353 (0.345; 0.370)
PSV^a^	persevering	5 (5; 5)	0.265 (0.267; 0.259)
REC^a^	comes when called (‘Recalls’)	5 (5; 5)	0.424 (0.466; 0.333)
SLP^a^	good at walking on slippery surfaces	5 (5; 5)	0.265 (0.302; 0.185)
SOC^a^	socially attached to you	5 (5; 5)	0.200 (0.224; 0.148)
STR^b^	nervous and tense when startled	1 (1; 1)	0.606 (0.552; 0.722)
TOY^a^	willing to give you a toy	5 (5; 5)	0.424 (0.457; 0.352)
WIL^a^	desires to make you happy (‘Willing to please’)	5 (5; 5)	0.353 (0.397; 0.259)

^a^Desirable descriptor.

^b^Undesirable descriptor.

^c^Brief descriptions used to form some abbreviations are shown in parentheses.

### 2.5. Network analysis

#### 2.5.1. Network construction

Networks were constructed and analysed using the *qgraph* package [54]. To construct networks that represented conditional independence relationships, we used Gaussian graphical models (GGM; see [55,56] for an overview). GGMs have been applied successfully to understand personality and psychopathology symptomatic networks (e.g. [27,57]). We used GGMs employing L_1_ lasso penalties (i.e. least absolute shrinkage and selection operator), where the inverse covariance matrix (i.e. the matrix of partial correlations) was subject to regularization through penalized maximum-likelihood estimation. This resulted in a sparse graph with credibly non-zero partial correlations, with partial correlations near zero being shrunk to zero. Regularization was controlled by a parameter λ∈[0,1] [58]. The optimal value of *λ* was chosen according to the graph with the lowest Extended Bayesian Information Criterion (EBIC) following Foygel & Drton [59] (see also [56]) and implemented in the ‘EBICglasso’ function in the *qgraph* package. The EBIC criterion was in turn tuned by a parameter γ∈[0,1] that performs best for positive values of *γ* [59]. We explored the networks over the entire range of *γ* (by 0.05 increments) and chose the most conservative value of *γ* = 0.65, where values above this resulted in empty graphs for the detection dog network. This method optimized specificity in network estimation (i.e. prioritized the elimination of truly non-existent edges) [60]. Because our data were ordinal, we conducted GGM construction and selection using the matrix of polychoric correlations (see the R script file in the electronic supplementary material), which provided the correlations between ordinal variables assumed to have latent continuous distributions.

#### 2.5.2. Centrality analysis

We explored and compared the structures of patrol and detection dog networks using node-level centrality metrics because nodes that are more central are more important for influencing network structure than peripheral nodes. We chose the metrics *betweenness* and *strength* centrality (defined formally for weighted networks in electronic supplementary material, table S3), where node betweenness represents how many shortest paths (i.e. with minimum distance between two nodes) run through a given node and node strength indicates how strongly each node is connected to other nodes [61,62]. Nodes with high betweenness values acted as mediators between indirectly connected nodes, and nodes with high strength values had stronger correlations with other descriptors.

#### 2.5.3. Network comparison and stability

To compare descriptor centrality between patrol and detection dog networks, 2000 non-parametric bootstrap samples for each network were computed (R package: *bootnet*) [63]. Each bootstrap constructed a network of randomly sampled dogs, with replacement. From these bootstrap samples, we calculated the mean centrality of each descriptor (the overall mean of descriptors' mean betweenness and strength values) and these means were compared with Cliff's delta (*δ*; R package: ‘*effsize*’) [64], a non-parametric effect size ranging between −1 and +1 (see [27]). To explore network stability, we computed bootstrap samples of the networks 2000 times from networks of 3 to 19 nodes (node-wise bootstrapping), and 2000 times from 25% to 95% (at approximately 8% increments) of the original sample sizes (subject-wise bootstrapping; see [65]). This allowed investigating the rank-order consistency of descriptor centrality values and the correlation between centrality values in the bootstrapped networks with the original networks. Confidence intervals on bootstrapped parameters are not reported due to known biases in their estimation [65].

## 3. Results

### 3.1. Descriptive network structures

**Figure 1.**
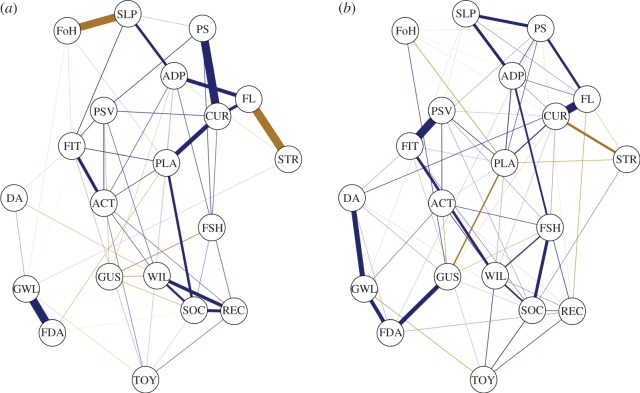
Gaussian graphical models of patrol (*a*) and detection (*b*) dogs. Blue edges show positive correlations, gold edges negative correlations; stronger correlations have thicker edges. See table 1 for descriptor abbreviations.

The patrol dog network (figure 1*a*; see association matrix in the electronic supplementary files) had 55 edges (28.95% of possible edges). ‘Curious’ had strong positive correlations with ‘Playful’, ‘Problem solving’ and ‘Fearless’. Additional salient positive correlations appeared between: ‘Socially attached to you’, ‘Recalls’ and ‘Willing to please’; ‘Strong tendency to growl at strangers’ and ‘Food aggressive’; ‘Good at walking on slippery surfaces’ and ‘Physically fit’; ‘Active and nimble’ and ‘Physically fit’; and ‘Fearless’ and ‘Adapts to new situations quickly’. Negative correlations were evident between: ‘Fearless’ and ‘Nervous and tense when startled’; ‘Fear of heights’ and ‘Good at walking on slippery surfaces’; ‘Dog aggressive’ and ‘Willing to please’; ‘Food aggressive’ and ‘Playful’; and ‘Gives up searches quickly’ with ‘Able to stay focused during searches’ and ‘Willing to please’.

The detection dog network (figure 1*b*; see association matrix in the electronic supplementary files) had 70 edges (36.84% of possible edges). ‘Playful’ shared salient positive correlations with ‘Curious’, ‘Persevering’, ‘Adapts to new situations quickly’ and ‘Problem solving’, and was most negatively correlated with ‘Gives up searches quickly’. ‘Able to stay focused during searches’ shared salient positive correlations with ‘Socially attached to you’, ‘Willing to please’, ‘Adapts to new situations quickly’, ‘Willing to give you a toy’ and ‘Active and nimble’. Strong positive correlations were also evident between: ‘Fearless’ and ‘Curious’; ‘Fearless’ and ‘Problem solving’; ‘Good at walking on slippery surfaces’ with ‘Problem solving’ and ‘Adapts to new situations quickly’; ‘Persevering’ and ‘Physically fit’; ‘Willing to please’ and ‘Socially attached to you’; ‘Gives up searches quickly’ and ‘Food aggressive’; and ‘Strong tendency to growl at strangers’ with ‘Food aggressive’ and ‘Dog aggressive’. A strong negative correlation was present between ‘Curious’ and ‘Nervous and tense when startled’.

### 3.2. Network centrality

**Figure 2.**
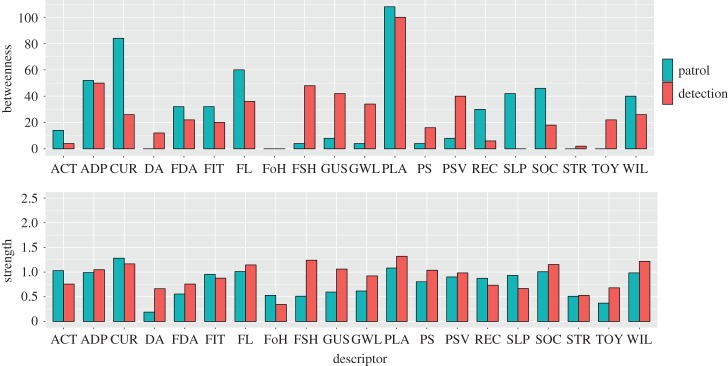
Observed betweenness and strength centrality values (bar heights) for patrol and detection dog networks. See table 1 for descriptor abbreviations and electronic supplementary material, table S4, for raw values.

Most of the desirable descriptors (table 1) had higher observed centrality values compared with undesirable descriptors (figure 2; see electronic supplementary material, table S4, for raw values). In the patrol dog network, ‘Playful’ had the highest betweenness centrality and ‘Curious’ the highest strength centrality, whereas ‘Playful’ had both the highest betweenness and highest strength centrality values in the detection dog network. Across both networks, ‘Active and nimble’, ‘Curious’, ‘Physically fit’, ‘Recalls’ and ‘Good at walking on slippery surfaces’ had higher betweenness and strength values in the patrol dog compared to detection dog network (figure 2). In the detection dog network, ‘Dog aggressive’, ‘Able to stay focused during searches’, ‘Gives up searches quickly’, ‘Strong tendency to growl at strangers’, ‘Problem solving’, ‘Persevering’, ‘Nervous and tense when startled’ and ‘Willing to give you a toy’ had higher betweenness and strength values than in the patrol dog network.

**Figure 3.**
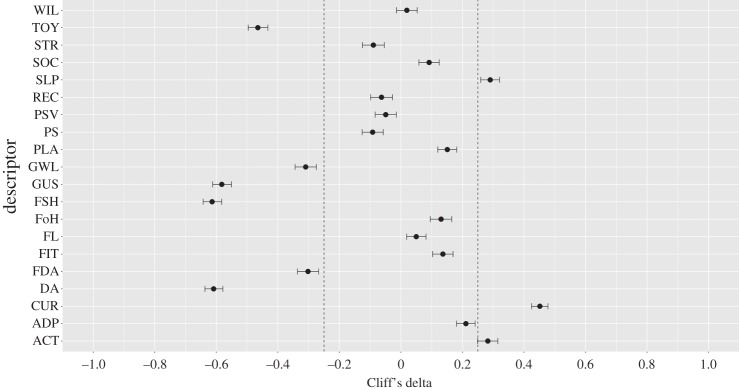
Cliff's delta effect sizes (and 95% CIs) for differences between patrol and detection dog centrality values (average of betweenness and strength) calculated from non-parametric bootstrap samples. Positive values indicate a larger mean for patrol dogs and negative values a larger mean for detection dogs. Values lying within the dashed lines at ±0.25 indicate negligible effect sizes. See table 1 for definitions of descriptor abbreviations and electronic supplementary material, table S5, for raw values.

Across non-parametric bootstrap samples, only certain descriptor centrality differences had strong effect sizes (figure 3; raw values provided in electronic supplementary material, table S5). Mean centrality differences in ‘Curious’ (*δ* = 0.452), ‘Good at walking on slippery surfaces’ (*δ* = 0.290) and ‘Active and nimble’ (*δ* = 0.282) had the largest effect sizes in favour of the patrol dog network. ‘Able to stay focused during searches’ (*δ* = −0.614), ‘Dog aggressive’ (*δ* = −0.609), ‘Gives up searches quickly’ (*δ* = −0.582), ‘Willing to give you a toy’ (*δ* = −0.465), ‘Strong tendency to growl at strangers’ (*δ* = −0.310) and ‘Food aggressive’ (*δ* = −0.302) had the largest effect sizes in favour of the detection dog network.

### 3.3. Network stability

**Figure 4.**
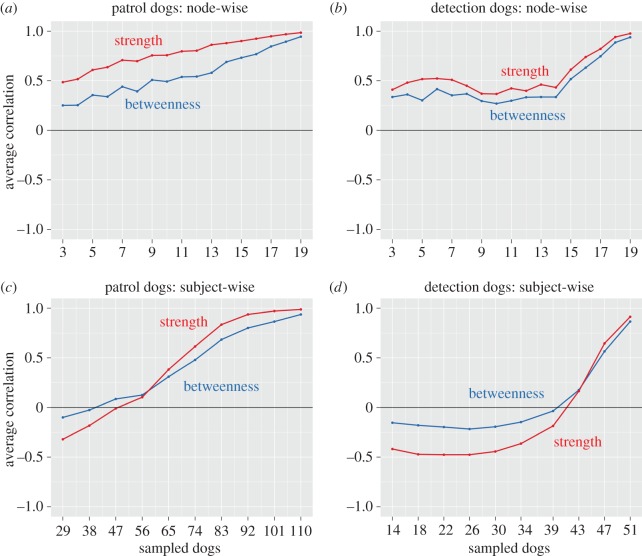
Stability of betweenness and strength centrality values in node-wise (*a,b*) and subject-wise (*c,d*) bootstrapping. The centrality values in each bootstrapped network were correlated with values in the original networks. Panels (*a*–*d*) show the average correlation across descriptors for each node-wise and subject-wise bootstrap sampling level, respectively.

The standard deviation of the number of edges in the patrol dog network across non-parametric bootstrap samples was 12.82, and 29.75 for the detection dog network. Node-wise bootstrapping demonstrated reasonable stability of the original network structures: centrality values from the bootstrapped networks were positively correlated with centrality values in the original networks (figure 4*a,b*), even for networks of only three nodes, although the patrol dog network was more stable than the detection dog network (see electronic supplementary material, figure S1, for the rank-order stability of individual descriptors). Network structure was more sensitive under subject-wise bootstrapping. For the patrol dog network, sampled networks of around 60 dogs or less (approximately 50% of the original sample size) showed little correlation with the original network values (figure 4*c*). For the detection dog network, networks less than around 40 dogs (approximately 70% of the original sample size) had low to negative correlations with the original network (figure 4*d*; see electronic supplementary material, figure S2, for the rank-order stability of individual descriptors).

## 4. Discussion

There has been much interest in biology about phenotypic integration of morphological traits, particularly their genetic and developmental bases [2,12,36]. Recent work has extended these notions to conceiving of the behavioural phenotype as composed of quasi-independent latent behavioural traits that form an integrated unit [7]. Network analysis offers benefits for understanding phenotypic integration [6,10,11] and has emerged in human psychology as an efficacious theoretical and analytical framework to understand human behaviour as a causally connected unit [17,32,33,66]. In this regard, it assimilates the study of behavioural phenotypes with research on a number of other complex systems showing how structure can emerge from self-organizing interactions between component parts (e.g. social groups [67], genetic/physiological networks [15,68] and evolutionary processes [14]). In this paper, we have assimilated this cross-disciplinary progression of ideas by using network analysis to understand relationships among behavioural and motivational characteristics in police patrol and detection dogs.

Our analyses revealed numerous direct correlations between functionally related descriptors in both patrol and detection dog networks (figure 1). For instance, behaviours related to aggression (‘Dog aggressive’, ‘Strong tendency to growl at strangers’ and ‘Food aggressive’) were positively correlated, especially in the detection dog network, as were descriptors indicating levels of sociability and/or trainability (‘Socially attached to you’, ‘Willing to please’, ‘Recalls’, ‘Willing to give you a toy’). Moreover, various positive correlations involving ‘Playful’, ‘Curious’, ‘Fearless’ and ‘Socially attached to you’ are partly consistent with Svartberg & Forkman's [39] interrelated factors ‘playfulness’, ‘curiosity/fearlessness’ and ‘sociability’. Together with ‘chase proneness’, these factors formed a super-trait referred to as ‘boldness’ that was related to working dog performance [44]. Svartberg & Forkman's [39] results were based on first- and second-order exploratory factor analyses of pairwise correlations, positing boldness as a higher-order latent variable causing covariation between boldness-related behaviours. Our findings extend these results by disentangling potential causal, mutually reinforcing relationships between behaviours. For instance, despite ‘Curious’ and ‘Socially attached to you’ sharing positive pairwise correlations (0.41 and 0.40 for patrol and detection dogs, respectively; see the R script file in the electronic supplementary files for their calculation), they were not directly related in either network of conditional independence relationships, suggesting that their pairwise correlation was due to common mediating variables. For the assessment of dog behaviour, low predictive values of behavioural and personality tests [41–43] may arise from over-estimating the homogeneity of behavioural traits from pairwise correlations when, in fact, trait compositions could be dynamic through time and across contexts. Distinguishing causal relationships from pairwise correlations could refine behavioural assessments through identifying behavioural variables that cause widespread changes in behavioural phenotypes.

Network analysis provides a number of unique metrics to understand patterns of relationships in multivariate data, such as the estimation of network centrality, indicating the relative importance individual components have across network topologies. In particular, the descriptor ‘Playful’ held a central position across networks (figure 2) both in its number of direct behavioural correlations (i.e. strength centrality) with other descriptors, but also in its mediating role between other relationships across the network (i.e. betweenness centrality). Playfulness is postulated to have a positive influence on the success or trainability of working dogs [44,45], comprising part of Svartberg & Forkman's boldness dimension [39], and has been assayed in working dog assessments by rating a dog's attentiveness and intensity when engaging in tug-type games with a toy [39,69]. Play also represents a heterogeneous category of behaviour that includes object-related, locomotory and social components [70], and constitutes an important method of reinforcement in training protocols. Thus, from a network viewpoint, playful behaviour may have important causal connections to a wide range of behaviours. In the patrol dog network (figure 1*a*), ‘Playful’ connected additional central descriptors (figure 2), such as between ‘Socially attached to you’ and ‘Curious’ or ‘Fearless’, respectively. In the detection dog network (figure 1*b*), ‘Playful’ had a strong negative relationship with ‘Gives up searches quickly’, the latter being particularly undesirable for detection dogs. As Bradshaw *et al.* [71] review, play in dogs correlates with a number of variables indicating positive well-being, including obedience indicative of close social bonds with owners. Therefore, the centrality of the ‘Playful’ descriptor in our network analyses holds an interesting organizational position in the behavioural phenotype of police dogs. This organizational role could be further examined in a network framework by quantifying how different forms of playful behaviour relate to other behaviours through time, or between breeds or types of dogs differing systematically in playfulness (e.g. working and pet dogs) [45].

Other descriptors differed in relative centralities between patrol and detection dog networks. In particular, ‘Curious’ had larger betweenness and strength centrality values in the patrol dog compared with the detection dog network (figure 2), which was also borne out in the non-parametric bootstrap analyses (figure 3). Moreover, ‘Good at walking on slippery surfaces’ and ‘Active and nimble’ had larger mean centrality values across bootstrap samples in the patrol dog compared to detection dog networks. By contrast, task-specific descriptors such as ‘Able to stay focused during searches’ and ‘Gives up searches quickly’ (which was negatively correlated with desirable descriptors such as ‘Playful’; figure 1*b*) were more central in the detection dog network than the patrol dog network, as was ‘Willing to give you a toy’, which may reflect the tendency for detection dogs to be trained to hold objects gently in their mouths and relinquish objects easily. Descriptors related to aggression were more frequently and strongly negatively correlated with desirable descriptors and positively correlated with undesirable descriptors compared to the patrol dog network. At the same time, weak positive correlations appeared between desirable and undesirable descriptors, such as between ‘Dog aggressive’ and ‘Fearless’, ‘Recalls’ and ‘Food aggressive’ or ‘Socially attached to you’ and ‘Nervous and tense when startled’, which were not present in the patrol dog network. These findings may indicate less stringent behavioural selection criteria for detection dogs compared with patrol dogs, conditional on detection dogs being good at searching. Consequently, successful detection dogs may, on average, be more likely to show correlations between undesirable and desirable behaviours than successful patrol dogs, as long as they show good performance during search tasks.

Nonetheless, our results also demonstrate uncertainty in network structures. Across non-parametric bootstrap samples, the detection dog network had a large standard deviation of estimated edges, probably due to the smaller detection dog sample size. Both networks were relatively stable in response to node-wise bootstrapping (figure 4*a,b*; electronic supplementary material, figure S1), but their stability was more sensitive in the subject-wise bootstrapping (figure 4*c*,*d*; electronic supplementary material, figure S2), and so may differ at larger samples sizes. As highlighted by Epskamp *et al.* [65], it is important that network analyses are checked for stability, and that uncertainty in parameter estimates is reported to gauge the predictive accuracy of network models. This is particularly important in dog personality studies employing exploratory analyses of multivariate datasets.

### 4.1. Limitations and future directions

There are potential limitations to the example presented here. First, the survey descriptors analysed include general behavioural and motivational characteristics (e.g. ‘Fearless’) that integrate a number of possible behaviour patterns. Thus, this lexical rating approach differs from the quantitative behavioural assays common in, for instance, behavioural ecology research. Nonetheless, rating approaches may be comparable or more beneficial than direct behavioural observations (e.g. in dogs: [72–74]), particularly in cases where raters are highly familiar with the individual animals (see also the discussion in [75]). However, while the survey here was completed by knowledgeable participants and explicated the network approach, no checks of reliability or validity were conducted. Instead, we employed a rigorous data cleaning process, removing 23 of the original 43 descriptors and employing multiple imputation of missing data. Checks of validity have not been fully developed under a network approach [76]. Validity theory attempts to answer whether an indicator measures what it is intended to measure (e.g. whether ‘Strong tendency to growl at strangers’ measures aggression) and is motivated by a ‘reflective’ latent variable conceptualization of scientific constructs [77]. However, the network approach does not view indicators, such as the behavioural descriptors analysed here, as measures of latent traits. Instead, the relationship between constructs and indicators is mereological [32,78], such that ‘the observables [i.e. indicators] do not measure the construct, but are part of it’ [32, p. 5]. Although validity in a network framework is currently in its infancy, exploring how the network approach can refine the predictive validities of current personality tests in dogs would be a fruitful avenue of research.

Secondly, the network analysis reported here was based on one survey per dog. Although handlers responded regarding dogs’ typical behaviours, there are advantages to gathering repeated measurements to directly estimate variation between and within individuals. Network analysis can also be applied to this end (e.g. see [79] for a multilevel time-series network model).

Finally, there is a natural relationship between integration of behavioural phenotypes and the study of animal personality and, relatedly, behavioural syndromes. Animal personality is defined by repeatable between-individual differences in behaviour reflecting personality traits [8,80,81]. As in studies of human personality, investigations into animal personality have used latent variable approaches (e.g. exploratory factor analysis [38] or structural equation modelling [72,82] in dogs) to extract relevant traits. However, the conceptualization of personality traits has been a point of confusion in animal behaviour [40,75,83] and psychologists have related a similar ambiguity in human research directly to latent variable interpretations [20,21,23,24]. Combining the network perspective established in human psychology and the more general biological concept of phenotypic integration may improve the clarity of personality definitions. That is, the behavioural phenotype becomes organized through causal connections between its components. By virtue of this organization, consistent behavioural expression is maintained through principles of network stability [84]. In this way, traits are emergent properties of clustering between functionally related behaviours [17,32]. In psychology, dynamic systems approaches to behaviour have a long history [85], supporting the process of behavioural integration as a self-organizing system [86,87]. In evolutionary biology, morphological trait complexes have been elucidated as emergent properties (‘evolutionarily stable configurations’) [12] and, more recently, Watson *et al.* [14] use principles of supervised and unsupervised learning to outline how phenotypic correlations can become causal connections over evolutionary timescales, highlighting the role of self-organization in the evolution of phenotypic integration.

## 5. Conclusion

Network analysis provides a novel approach to conceptualizing and analysing the behavioural phenotype, in both humans and animals. Following recent work across the biological study of phenotypic integration and human psychology, network analysis can be used to conceive of the behavioural repertoire of individuals as a connected system of causally dependent components. We have demonstrated how network analysis can be applied using police patrol and detection dogs as an example, elucidating commonalities and differences between networks in the interrelationships between behavioural and motivational descriptors. Moreover, we have demonstrated how analyses can be carried out to ascertain the stability of the results. We conclude that a network approach offers widespread opportunities for advancing the understanding of phenotypic integration in animal behaviour.

## Supplementary Material

Supplementary Material

## Supplementary Material

‘Supplementary Material’ This document contains details about the statistical analyses not included in the main text, extra descriptive statistics and results of the network analyses

## Data accessibility

The electronic supplementary material and the electronic supplementary files (including the complete raw data, the data after multiple imputation, the patrol and detection dog Gaussian graphical model association matrices, and R script file to reproduce the analyses and figures in this article) are available on Dryad Digital Repository at http://dx.doi.org/10.5061/dryad.81k11 [88].
