# Supplementary material for: Using network analysis to study behavioural phenotypes: an example using domestic dogs
Source: R Soc Open Sci. 2016 Oct 19;3(10):160268. doi: 10.1098/rsos.160268 (PMC5098969; doi:10.1098/rsos.160268)
Supplement: Supplementary Material [file rsos160268supp1.pdf]

Supplementary Material

This document contains the Supplementary Material for the article:
Goold C., Vas J., Olsen C., Newberry RC. "A network approach to integrating behavioural phenotypes: an example using domestic dogs". Supplementary Files including the data and R script also provided on Dryad Digital Repository at http://dx.doi.org/10.5061/dryad.81k11.

Table/Figure	Page
**Section 1.** Determining potential biases in the pattern of missingness (before multiple imputation)	1
**Section 2.** Removal of highly correlated descriptors	2
**Section 3.** Determining data independence	2
**Table S1.** Full list of descriptors, with missing and zero responses	3
**Table S2.** Likelihood ratio test results	5
**Table S3.** Formal definitions of node centrality metrics	6
**Table S4.** Raw centrality values for each network	7
**Table S5.** Cliff's Delta effect size results	8
**Figure S1.** Stability of centrality values across node-wise bootstrapping	9
**Figure S2.** Stability of centrality values across subject-wise bootstrapping	10

Section 1

Statistical details on determining potential biases in the pattern of missingness in the data prior to multiple imputation.

To understand whether missingness (Table S1) was dependent on dog type, we used a generalised linear model with logit link modelling each dog's relative number of missing values, y_i = P(Number missing | Number not missing), as an influence of a dichotomous fixed effect indicating patrol or detection dog status. Patrol dogs had a significantly smaller proportion of missing values compared to detection dogs (detection dogs = 2.63%; patrol dogs =1.11%; β = - 0.88; SE = 0.2; z = -4.41; p < 0.001). To determine whether missingness varied between handlers filling out more than one survey on different dogs, we used a generalised linear mixed model (using the *lme4* package; Bates et al. 2015) with logit link, only including the 44 handlers with repeated responses, with a fixed effect of handler type (whether they owned detection or patrol dogs) and a random intercept for handler ID. We calculated the amount of between- to within-handler variance in the relative number of missing values using the intraclass correlation coefficient (ICC, i.e. variance in random

intercept over complete variance), with the residual variance set to $\pi^2/3$ (Nasagawa & Shielzeth, 2010). The ICC was 0.158, indicating 15.8% of the variance in the proportion of missing responses could be attributed to between-handler differences.

Bates D, Maechler M, Bolker B, Walker S. 2015. Fitting linear mixed-effects models using lme4. J Stat Soft 67, 1-48. doi: 10.18637/jss.v067.i01.

Nakagawa S, Schielzet H. 2010. Repeatability for Gaussian and non-Gaussian data: a practical guide for biologists. Biol Rev 85, 935-956. doi: 10.1111/j.1469-185X.2010.00141.x.

Section 2

Removal of descriptors that were highly correlated with another descriptor of theoretical similarity.

We identified 5 pairs of variables that were theoretically similar and had high correlations relative to the data as a whole (polychoric correlations > | 0.8 |), indicating redundancy: i) 'Keeps balance on unstable surfaces' and 'Good at walking on slippery surfaces', ii) 'Stranger aggressive' and 'Strong tendency to growl at strangers', iii) 'Quick' and 'Active and nimble', iv) 'Confident in unfamiliar places' and 'Adapts quickly to new situations', and v) 'Obedient' and 'Comes when called'. We removed the first descriptor of each pair (Table S1).

Section 3

Statistical details on determining independence of responses by handlers filling out surveys for more than one dog.

For each question, we subsetted the data for those 44 handlers with repeated responses and computed ordinal cumulative link regression models with logit links (using the *ordinal* package; Christensen, 2015), with a fixed effect of handler type (i.e. whether handlers had patrol or detection dogs) and either with (the full model) or without (the simpler model) a random intercept for handler ID. We compared these models by the change in Akaike's Information Criteria ($\Delta AIC = AIC^{full} - AIC^{simpler}$) and likelihood ratio tests. The latter tests whether the ratios of the models' log-likelihoods are significantly different from zero ($\alpha = 0.05$). When $\Delta AIC \leq -2$ the full model was considered a better explanation of the data generating process compared to the simpler model, i.e. the between-handler variation was large enough to consider repeated responses as non-independent. These analyses resulted in a further 8 descriptors being removed (Tables S1-S2).

Christensen RHB. 2015. ordinal – Regression models for ordinal data. R package version 2015. 6-28. https://cran.r-project.org/web/packages/ordinal/index.html.

Table S1. Full list of desirable and undesirable behaviour descriptors in police dog handler survey, in the original order of presentation. Descriptors preceded by Roman numerals were removed before the network analyses (see footnotes for details).

Abbreviation	Full Descriptor	*n* = missing	*n* = zeros
[I]COU[1]	Courageous when threatened by a person	4	24
[II]CB[1]	Good at catching a ball	1	0
[III]ACO[1]	Able to carry an object	5	5
[IV]FHO[1]	Finds hidden objects easily	1	2
SLP[1]	Good at walking on slippery surfaces	3	0
[V]BAL[1]	Keeps balance on unstable surfaces	3	1
PS[1]	Solves problems on own (‘Problem solving’)	2	0
PSV[1]	Persevering	0	0
FL[1]	Fearless	1	0
CUR[1]	Curious	1	0
PLA[1]	Playful	0	0
FIT[1]	Physically fit	1	0
SOC[1]	Socially attached to you	2	0
DA[2]	Aggressive towards other dogs (‘Dog aggressive’)[3]	0	0
[V]SA[2]	Aggressive towards unfamiliar people (‘Stranger aggressive’)	0	4
GWL[2]	Strong tendency to growl at strangers	2	5
[V]OBD[1]	Obedient	0	0
REC[1]	Comes when called (‘Recalls’)	1	0
[IV]OCD[1]	Obeys commands when unfamiliar dogs are present	0	0
[IV]OCN[1]	Willing to obey commands in a noisy environment	1	1
[V]QK[1]	Fast (‘Quick’)	2	0
ACT[1]	Active and nimble	1	0
[V]CON[1]	Confident in unfamiliar places	0	0
ADP[1]	Adapts quickly to new situations	0	0
[IV]ANX[2]	Anxious when separated from handler in unfamiliar places	0	3
STR[2]	Nervous and tense when startled	2	5
[IV]QU[1]	Quiet – doesn’t bark much	0	0
[I]COH[1]	Willing to cooperate with other handlers	0	18
FSH[1]	Able to stay focused during searches	3	0

GUS[2]	Gives up searches quickly	1	1
[III]ODR[1]	Able to recognize odours	3	9
[III]FOV[1]	Able to find objects using vision	3	6
[I]AQS[1]	Able to hear quiet sounds	0	17
[IV]TC[2]	Chases own tail ('Tail chases')	0	4
[IV]CC[2]	Wants to chase cars	0	1
FDA[2]	Guards food ('Food aggressive')	0	3
TOY[1]	Willing to give you a toy	0	0
[I]BPH[1*]	Bites people hard	1	18
[IV]TUG[1]	Tugs hard in "tug-of-war" games	1	1
FoH[2]	Fear of heights	0	4
[I]DST[2]	Difficult to stop once starts an attack	3	37
[III]TT[2]	Tucks tail between legs	4	6
WIL[1]	Desires to make you happy ('Willing to please')	0	3

[1] Desirable descriptors
[1*] Desirability depended on context, resulting in ambiguous responses
[2] Undesirable descriptors
[3] Parentheses indicate shortened full descriptor formats used to form abbreviations
[I] Removed: more than 10% of 'Not relevant/I do not know' responses
[II] Removed: little variation after multiple imputation
[III] Removed: more than 5% of values to impute (i.e. missing + zero responses)
[IV] Removed: non-independence of repeated responses from the same handlers (see Table S2)
[V] Removed: possessing correlations > | 0.8 | with theoretically similar descriptors

Table S2. Likelihood ratio test results comparing full models (models with handler ID as a random intercept) and simpler models (models with no random effect). The ΔAIC = $AIC^{full} – AIC^{simpler}$, where ΔAIC ≤ -2 indicates the full model fits better and corroborates the *p* values. Bold fields show descriptors where the full model is preferred and, thus, these descriptors were removed from the main network analyses. In each test, the degrees of freedom = 1 (i.e. the models differ by one parameter).

**Descriptor**	**Δ AIC**	**Likelihood ratio statistic**	***p* value**
ACT	2	0	0.988
ADP	0.93	1.069	0.301
**ANX**	**-8.13**	**10.131**	**0.001**
CB	1.731	0.269	0.604
**CC**	**-4.85**	**6.85**	**0.009**
CUR	2	0	0.996
DA	0.34	1.66	0.2
FDA	-1.75	3.753	0.052
**FHO**	**-2.62**	**4.617**	**0.032**
FIT	0.17	1.822	0.177
FL	1.99	0.011	0.915
FoH	-0.62	2.626	0.105
FSH	0.1	1.9	0.168
GUS	1.69	0.31	0.578
GWL	-0.76	2.762	0.097
**OCD**	**-1.99**	**3.985**	**0.046**
**OCN**	**-8.46**	**10.46**	**0.001**
PLA	2	0.002	0.968
PS	1.97	0.024	0.877
PSV	1.99	0.009	0.927
**QU**	**-19.13**	**21.133**	**0**
REC	0.46	1.535	0.215
SLP	1.04	0.959	0.327
SOC	1.69	0.308	0.579
STR	-0.54	2.536	0.111
**TC**	**-3.68**	**5.68**	**0.017**
TOY	1.4	0.601	0.438
**TUG**	**-6.39**	**8.386**	**0.004**
WIL	-0.62	2.626	0.105

Table S3. Node centrality metrics.

**Metric**	**Calculation**	**Definition**
Betweenness	$C_b(i)=\sum_{s\neq i\neq t\in V}\frac{\sigma_{st}^{w}(i)}{\sigma_{st}^{w}}$	The betweenness centrality of node *i* equals the sum of shortest paths σ between nodes *s* and *t* of the set of all nodes *V* that travel through *i*, over all shortest paths between *s* and *t*. In weighted networks, where shorter path lengths may be superseded by longer but strongly connected paths, σ_{st} is defined as the minimum shortest paths between nodes $d^{w}(i,j)$, which is the path between nodes *i* and *j* passing through nodes *h*, that has the minimum sum of inverse edge weights or least 'cost' , represented here as σ_{st}^{w}.
Strength	$C_s(i)=\sum_{j=1}^{N}w_{ij}$	The strength C_s of node *i* equals the sum of the weights w_{ij} of edges *j* to *N* adjacent to *i*.

Table S4. Betweenness and strength centrality values for patrol and detection dog networks

Descriptor	Patrol betweenness	Detection betweenness	Patrol strength	Detection strength
ACT	14	4	1.026	0.758
ADP	52	50	0.990	1.049
CUR	84	26	1.282	1.168
DA	0	12	0.189	0.664
FDA	32	22	0.556	0.759
FIT	32	20	0.954	0.877
FL	60	36	1.011	1.146
FoH	0	0	0.528	0.345
FSH	4	48	0.511	1.240
GUS	8	42	0.596	1.062
GWL	4	34	0.617	0.922
PLA	108	100	1.083	1.321
PS	4	16	0.805	1.039
PSV	8	40	0.901	0.984
REC	30	6	0.873	0.736
SLP	42	0	0.932	0.667
SOC	46	18	1.008	1.155
STR	0	2	0.510	0.529
TOY	0	22	0.372	0.682
WIL	40	26	0.984	1.218

Table S5. Cliff's Delta effect size statistics, including 95% CI.

Descriptor	Mean effect size	Lower CI	Upper CI
ACT	0.282	0.249	0.315
ADP	0.211	0.181	0.241
CUR	0.452	0.425	0.478
DA	-0.609	-0.637	-0.579
FDA	-0.302	-0.336	-0.268
FIT	0.136	0.103	0.169
FL	0.050	0.019	0.081
FoH	0.130	0.095	0.165
FSH	-0.614	-0.643	-0.582
GUS	-0.582	-0.612	-0.551
GWL	-0.310	-0.344	-0.275
PLA	0.151	0.120	0.181
PS	-0.092	-0.126	-0.057
PSV	-0.049	-0.084	-0.015
REC	-0.063	-0.098	-0.027
SLP	0.290	0.259	0.320
SOC	0.092	0.059	0.124
STR	-0.089	-0.125	-0.054
TOY	-0.465	-0.496	-0.432
WIL	0.019	-0.014	0.053

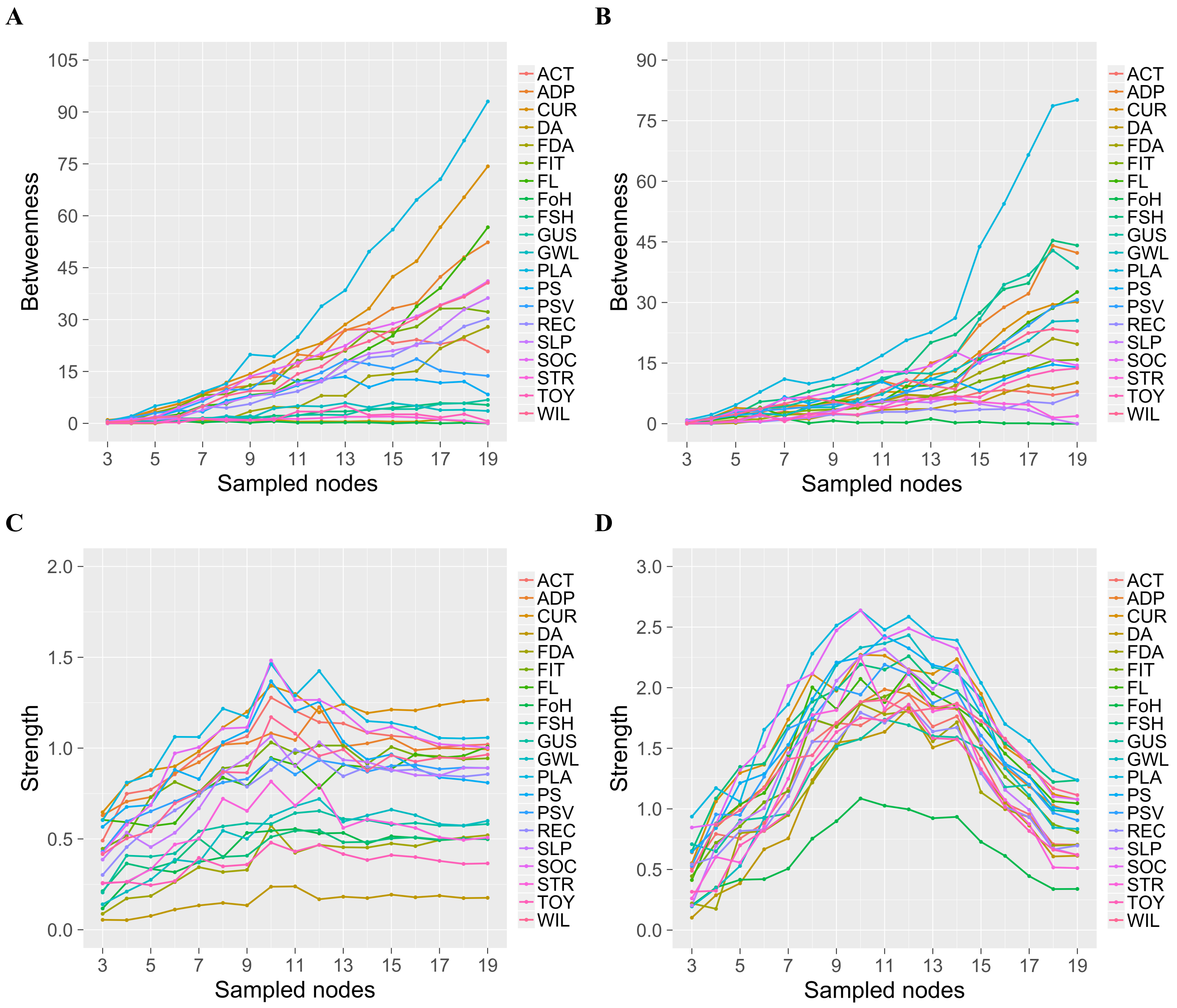

Figure S1. Rank-order stability of betweenness (A-B) and strength (C-D) centrality across node-wise bootstrap samples for patrol dogs (A & C) and detection dogs (B & D).

A

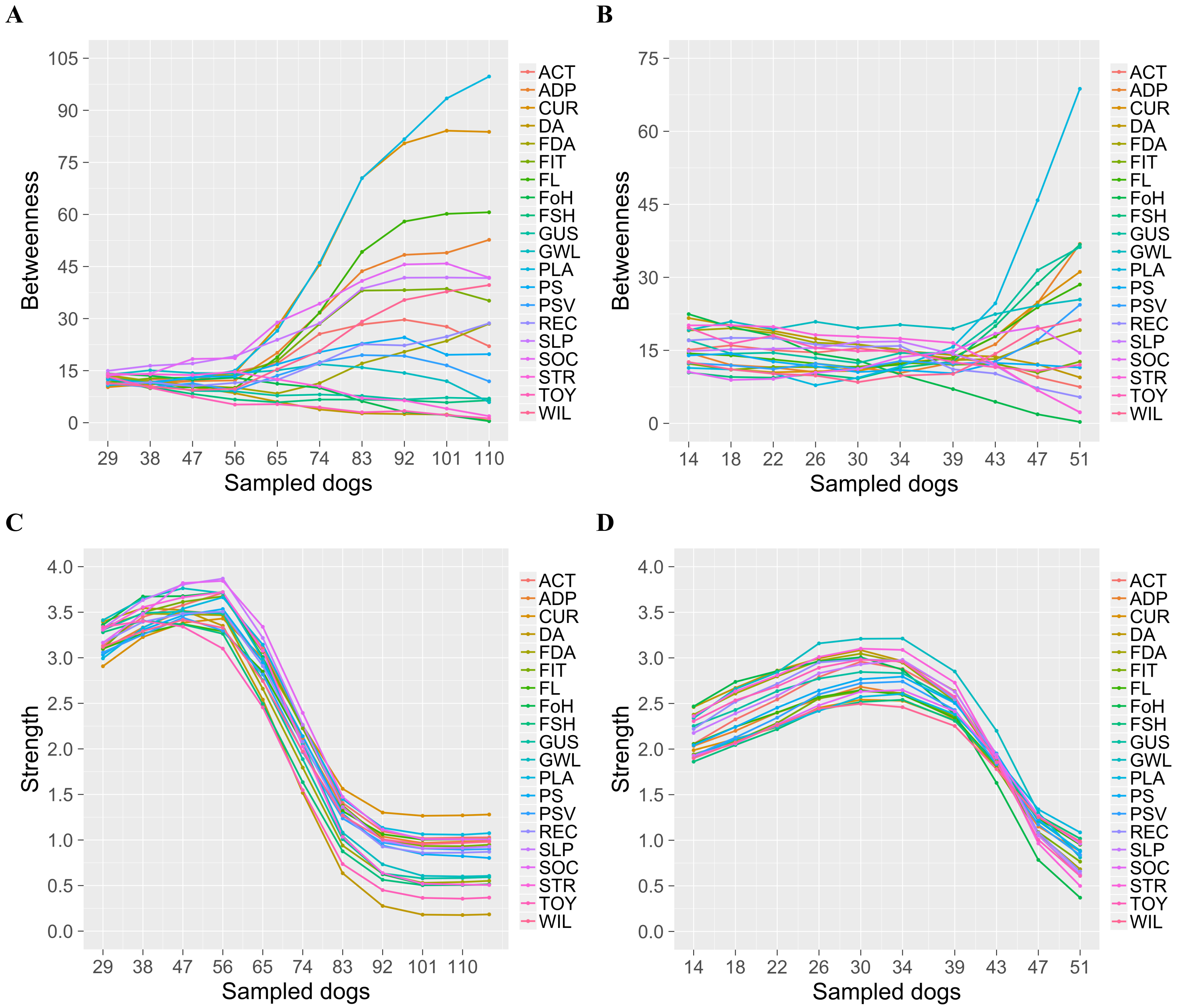

Figure S2. Rank-order stability of betweenness (A-B) and strength (C-D) centrality across subject-wise bootstrap samples for patrol dogs (A & C) and detection dogs (B & D).
